# More but Smaller: Marine Heatwaves Exacerbate Size Truncation in Overfished Fish Communities in the Skagerrak

**DOI:** 10.1002/ece3.71404

**Published:** 2025-05-08

**Authors:** Kjell Magnus Norderhaug, Portia Joy Nillos Kleiven, Thomas Wernberg, Ann‐Elin Synnes, Karen Filbee‐Dexter, Sigurd Espeland, Jonas Thormar, Kyrre Heldal Kartveit, Lene Christensen, Even Moland

**Affiliations:** ^1^ Institute of Marine Research (IMR) His Norway; ^2^ UWA Oceans Institute & School of Biological Sciences University of Western Australia Crawley Western Australia Australia; ^3^ Department of Natural Sciences, Centre for Coastal Research University of Agder Kristiansand Norway

**Keywords:** gadidae, gobies, kelp forest, labrids, marine habitats, turf

## Abstract

As we are progressing through the Anthropocene, it is imperative to understand how ecosystems degraded by historical impacts respond to future pressures. Particular attention should be directed at changes in the distribution and function of foundation species due to their disproportionately important ecological roles. Forest‐forming kelps are in decline and are being replaced by turf‐forming algae in coastal areas globally, as a consequence of increasingly severe marine heatwaves (MHW). This replacement represents a miniaturization of the three‐dimensional habitat and a change in habitat functions for many associated species. Ecologically, economically, and culturally important coastal fish species depend on kelp forests in different life stages from egg to adult and may be affected in various ways. Many of these species already suffer from centuries of overexploitation, severely reducing abundances, truncating sizes, and changing age distributions. Here, we studied an overfished coastal ecosystem and assessed the impact of the transition from kelp to turf on the structure and function of associated fish communities. Specifically, we analyzed differences in abundance, body size, and composition of benthic fish at sites with variable cover of kelp and turf. We found higher abundances of small fish with increasing cover of turf relative to kelp. We also found smaller fish within the common families of gobids and gadids. Thus, MHW exacerbate size truncation and trophic downgrading in coastal fish communities. However, no differences in abundances of fish in the water column were detected during the night, and the average size of fish at turf‐dominated sites was even larger. We interpreted this as roving predatory fish during night‐time being less affected by the shift in habitat and smaller‐bodied prey avoiding the water column in open turf habitats.

## Graphical abstract

Habitat forming kelps are in decline and are being replaced by turf‐forming algae in coastal areas globally due to increasing frequency and intensity of MHW. By favoring small fish species, these shifts exacerbate size distribution effects in coastal fish communities, with many stocks already suffering from overfishing.

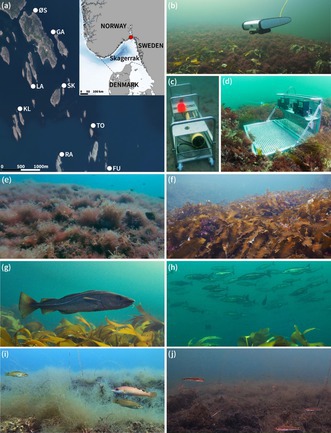

## 1 Introduction

Human activities are increasingly taking their toll on all ecosystems. The main drivers of impacts are, however, changing, and while hunting and harvesting historically have had the most severe impacts on marine life (Duarte 2014; Duarte et al. 2020; IPBES 2019), climate change is expected to be the primary threat for global biodiversity in the future (IPCC 2019; Wernberg et al. 2024). Habitat‐building species transform their environments and create living spaces and services for species inhabiting them (Bruno et al. 2003). Their disproportionately important roles to other species imply they are particularly valuable for maintaining biodiversity and ecosystem functions across all environments. In light of the biodiversity crisis acknowledged through the UN Convention on Biological Diversity (CBD, United Nations 1992), particular awareness should be given to the ecological and economic consequences of the change of these foundation species in nature management (Smith et al. 2024; Wernberg et al. 2024). It is therefore of great concern that habitats are in decline globally, both in terrestrial and, more recently, also aquatic environments (McCauley et al. 2015; Wernberg et al. 2024), and the loss of habitats is highlighted as one of the main threats to marine biodiversity in the future (Rogers et al. 2020; IPCC 2019).

Kelp are perennial marine algae forming dense forests in temperate shallow waters (Wernberg et al. 2023). They are important coastal habitats supplying food, sequestering carbon, and supporting high biodiversity, including commercially important fish stocks, such as goldsinny wrasse, pollock, haddock, and cod, as well as providing cultural services for humans (Wernberg et al. 2023; Eger et al. 2023). Fish use kelp forests for various purposes and in different parts of their life cycle. For example, kelp forests provide nesting areas for labrids and nursery areas for saithe and other gadoids (Potts 1985; Norderhaug et al. 2005), and they are feeding areas for fish ranging from small territorial labrids to large gadoid predators with large home ranges (Norderhaug et al. 2020; Freitas et al. 2021).

Global warming has emerged as a dominant driver for the redistribution of life on Earth, and it represents an imminent threat to kelp in many coastal areas (Pecl et al. 2017; Filbee‐Dexter and Wernberg 2018). The longest available time series suggests that almost 60% of the world's kelp forests have been in decline over the past decades (Wernberg et al. 2019). As a global phenomenon, in many places, turf‐forming algae comprised of filamentous algae replace kelp where these foundation species are lost (Filbee‐Dexter and Wernberg 2018). Skagerrak in the NE Atlantic represents the warm edge distributional range for the kelp *Saccharina latissima*, and substantial loss of kelp and replacement by turf algae following marine heatwaves (MHW) has been documented: Filbee‐Dexter et al. (2020) revealed a two‐ to four‐fold increase in intensity and occurrence of MHWs over a 60‐year period in the Skagerrak and eastern USA, coinciding with the disappearance of kelps on both sides of the Atlantic. They also demonstrated a relationship between the 2018 heatwave and high kelp mortality in both regions by in situ experiments. Consequently, the three‐dimensional forest habitat has been transformed into flattened turfs with different form and function. The transformation represents a miniaturization, homogenization, and reduction in habitat complexity (Pessarrodona et al. 2021). Algal habitat structure affects the functions of associated benthic fish communities (Tuya et al. 2009), but the consequences for the structure and function of associated fish communities when kelp is replaced by turf are largely unknown.

New genetic tools providing long‐term information on effective population sizes indicate that humans have impacted predatory fish stocks for centuries, far earlier than previously anticipated (Sodeland et al. 2022). Many fish stocks inhabiting kelp forests are already struggling from overexploitation in coastal areas globally (Jackson et al. 2001; Estes et al. 2011; Moore et al. 2024). Fishing of predatory fish has caused depletion and size truncation of targeted stocks, loss of resilience against drivers of change, and cascading perturbations through food webs leading to blooms of grazers and opportunistic algal species with consequent loss of habitat‐building macrophytes (Jackson et al. 2001; Moksnes et al. 2008; Wernberg et al. 2016). The Skagerrak coast is densely populated, and fishing has historically been an important component of the coastal population's food source, culture, and income. Naturally occurring large predatory fish, including sharks, tuna, Atlantic cod, and Atlantic wolffish, were either intensively targeted or by‐caught during the 20th and 21st centuries, and once productive local populations have been decimated or serially depleted (Sundelöf et al. 2013; Cardinale et al. 2017). In the 21st century, this coastal ecosystem can be characterized as almost emptied of large predators (Large Fish Indicator, Svedäng and Bardon 2003; Cardinale and Svedäng 2004; Sundelöf et al. 2013). Recently, Synnes et al. (2023) compared shallow water coastal fish assemblages in two Skagerrak coastal seascapes, in which one overlapped with the present study area. Their study pointed to the absence of large‐bodied piscivorous predatory fish species in the outer Oslofjord. Trophic downgrading was less pronounced in the Tvedestrand fjord seascape, which harbored a no‐take zone and partially protected areas implemented in 2012.

Increased knowledge regarding how ecosystems already impacted by historical drivers respond to climate change is vital for environmental management in the Anthropocene. The Skagerrak's history of overfishing in combination with documented loss of kelp due to increasing intensity and frequency of MHWs makes it well suited for research addressing this topic. In this study, we assessed if habitat flattening and miniaturization that occurs when forest‐forming kelp is transformed into turf impact associated fish communities in the Skagerrak, a coastal area already heavily overfished. Specifically, we analyzed differences in composition, abundance, and sizes of benthic fish during night and day in habitats with variable kelp and turf cover.

## 2 Methods

### 2.1 Sampling Area

**FIGURE 1:**
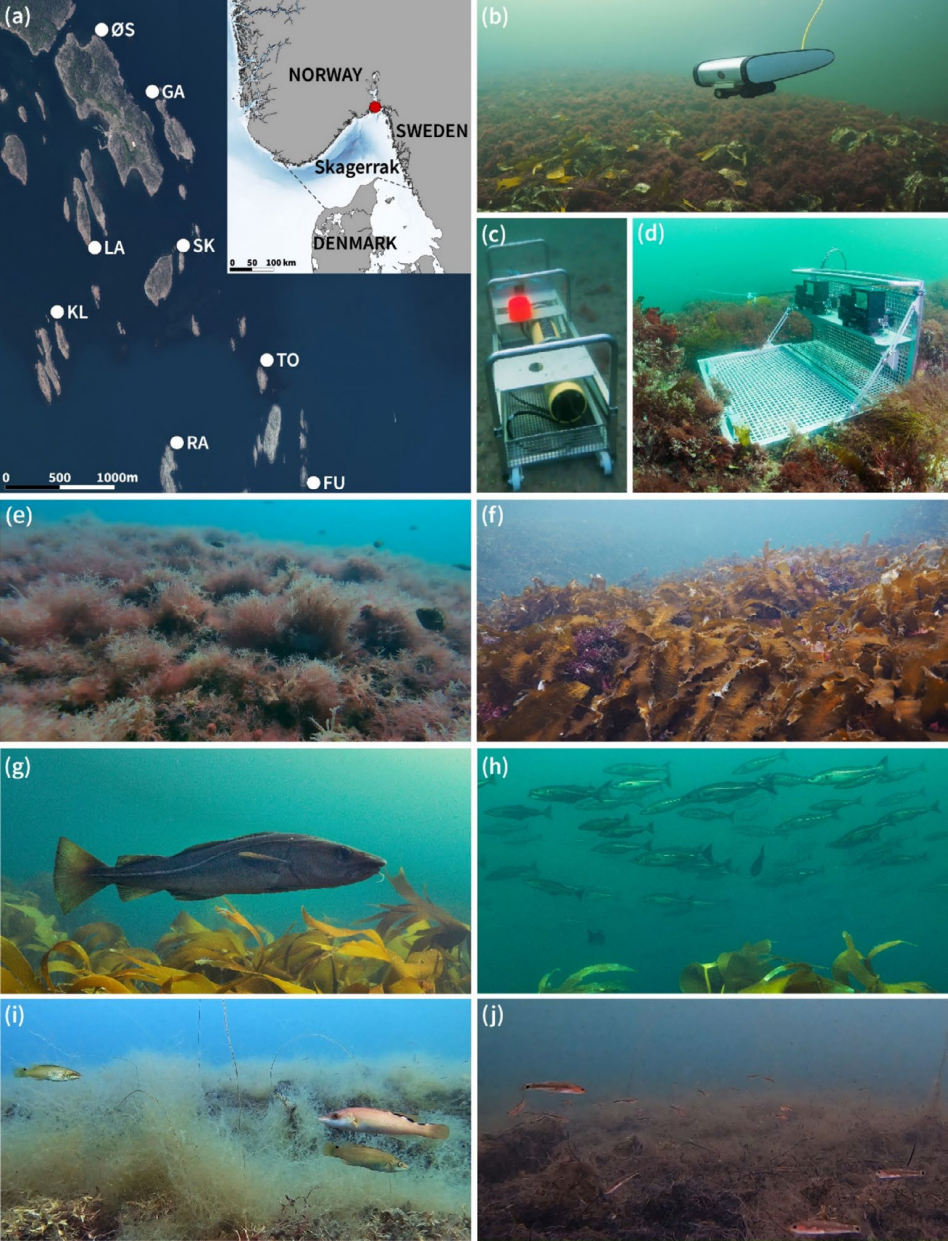
From upper left to lower right: (a) Sampling sites in the Bolærne archipelago in Skagerrak; sampling techniques: (b) Remotely operated vehicle (ROV), (c) bottom‐mounted autonomous scientific echosounder, WBAT, and baited remote underwater stereo video BRUV; Habitats: (e) turf bed, (f) kelp *Saccharina* forest; and fish: (g) gadids cod *Gadus morhua* , and (h) saithe *Pollachius virens*, (*i*) labrids *Ctenolabrus rupestris* and *Labrus mixtus*, and (j) two‐spotted goby *Pomatoschistus flavescens* (photos: J Thormar).

The Bolærne archipelago on the Skagerrak coast of Norway is characterized by small islands and a shallow rocky bottom suitable for kelp (Figure 1). Kelp forests with variable domination of *Laminaria* and *Saccharina* are mixed with areas where the formation of turf has more or less replaced kelp (Filbee‐Dexter et al. 2020). Eight sites, with varying coverage of kelp and turf and with otherwise similar environmental conditions and topography of sloping rocky substrate, were selected for the study (Figure 1, Appendix S1: Table S1). Sampling was performed during 10 to 14 August 2020 when sunrise was at 05:25 in the morning and sunset at 21:24 in the evening.

### 2.2 Sampling Methods

Methodological challenges apply to traditional, destructive fishing methods such as nets and traps when comparing data inside and outside dense vegetation. Because of different catchability and because they fail to catch small fish, the use of a combination of sampling tools is recommended to obtain representative data across different scales in space and time (Duffy et al. 2019). Baited remote underwater stereo video (BRUVs, Figure 1, Langlois et al. 2010) has proven to be a reliable method in achieving comparable data of fish body size from dense vegetation and open areas (Perry et al. 2018; Letessier et al. 2024). Bottom‐mounted upward‐facing echosounders collect data passively and independently of turbidity and visibility in dense vegetation and independent of light during night and day (Norderhaug et al. 2020). For assessing habitat cover, a remotely operated vehicle (ROV) equipped with a camera is useful for obtaining comparable data across sites (Duffy et al. 2019). All these methods used are non‐destructive.

### 2.3 Remotely Operated Vehicle (ROV)

An ROV with a camera was used to assess the vertical zonation of algae habitats at each site. ROV transects were run from 0 to 20 m depth (or maximum 100 m long transect) along a pre‐deployed sinking line to map the vertical zonation of vegetation at each site (Figure 1). Vegetation classified into kelp forest (*Saccharina latissima, Laminaria hyperborea* and *L. digitata* ), turf (various filamentous annual green, brown and red filamentous algae creating a carpet on the sea floor, Pessarrodona et al. 2021), or no vegetation were measured as % cover. Each site showed variable cover of kelp and turf ranging from 0 to 12–17 m depth (Appendix S2: Figure S1). At Garnholmen and Langholmen, turf cover was almost complete, while complete kelp cover was found at Fulehuk and Rauer. The other sites had variable covers of kelp and turf. In figures and statistical analysis, the cover of turf and kelp, respectively, were combined into one metric by dividing % cover of turf by % cover of kelp (turf: kelp ratio).

### 2.4 Baited Remote Underwater Stereo Video BRUV

Fish were assessed by baited remote underwater stereo video (BRUV) rigs deployed during day (before sunset) and night (after sunset, with light), at 5–7 m and 10–15 m depth at each site to include fish at different times and depths (Figure 1, Appendix S1: Table S1). Stereo video provides depth vision and makes it possible to assess the abundance of fish in a defined and limited water volume (Denney et al. 2017). Since BRUVs are baited (with 200 g mackerel, Jones et al. 2020) they primarily register feeding fish. For each deployment, 1 h of video footage was analyzed. The MaxN (the maximum number of fish observed in any given rotation of the camera system) was calculated for each rig and a representative number of fish lengths measured using the EventMeasure software. Fish lengths could not be calculated for the deepest night‐time deployments at Langholmen and Skarvesetet because only one camera functioned. The deepest deployments from Skarvesetet during day and Rauer during night are missing (NA in Table 2) due to upright‐facing cameras preventing the observer from identifying fish correctly. Since BRUVs were baited, WBAT and BRUVs were not deployed simultaneously at the same site.

### 2.5 WBAT Autonomous Scientific Echosounders

Two bottom‐mounted and upward facing autonomous scientific echosounders were used to assess fish diurnal activity. Simrad WBAT EK80 autonomous scientific echo sounder units (https://www.kongsberg.com/maritime/products/ocean‐science/ocean‐science/es_scientific/wbat/) with 200 kHz CW (ES200‐7CD) transducers were used for acoustic assessments of diurnal variation of fish abundance. The units are upward facing and were placed at 10 m depth under the observation of a SCUBA diver. Kelp laminas immediately over the transducer were removed by the diver to avoid disturbances. They were set to record continuously between 20.00 (before sunset) and 08.00 (after sunrise) at each site (Figure 1, Appendix S1: Table S1). The units were run with equal settings in every deployment (ping interval: 0.25 s, beam type: split, power: 75, pulse type: CW, start frequency: −200 kHz, pulse duration: 256 μs, ramping: Fast, range: 15–20 m, TX mode: Active) and were CW calibrated with Tungsten Carbide (38.100 mm nominal diameter) spheres from Redhill. Fish was defined as echoes with target strength −60 dB, which is the target strength of e.g., a 4 cm long sprat, to −20 dB (Simmonds and MacLennan 2008). Fish abundances (n/m^3^) and size (target strength [TS] value) are assumed to be reflected in target strength. TS increases with the size of a fish but also with other factors like shape and orientation of the target, in this case the swim bladder of the fish (which is the dominant reflecting organ) (Simmonds and MacLennan 2008). Furthermore, the relationship between TS and fish size is dependent on the fish species (Foote et al. 1986; McClatchie et al. 1996), but as this study is focused on the presence and abundance of fish, we have not utilized species‐specific models to relate TS to size. It is also possible to address the effect of fish orientation by examining the trajectory of tracked fish, but as this falls outside the scope of this paper, this has not been investigated further. For a thorough description of this relationship, the reader is referred to (e.g., Mann et al. 2008; Stanton et al. 2003). In this study, all registered fish (except a few mackerel) had swim bladders, and TS was thus generally considered representative for fish size.

### 2.6 Statistical and Numerical Methods

We used generalized linear models (GLM) to determine the response of variables (total abundance of the most abundant families, gadids, labrids, gobies, as well as numbers detected by WBAT and MaxN observed with EventMeasure) to the varying substrate conditions. GLM was used because the variance of the response variables was not constant and none of them were normally distributed. We formulated a GLM using MaxN (abundance) as the response to the turf and kelp ratio for (*a*) the three most abundant and most commonly occurring families (gobids, gadids and labrids).

Outliers (those that had an unusually high MaxN of over 100 individuals) were inspected to see if they were potential outliers from the data set before running GLM. We used the following basic model structure:

$${ℽ}_{i}\sim \text{Poisson}\;\left(\mu_{i}\right)$$

$$\log\left(\mu_{i}\right)=\alpha +\beta_{\mathrm{xi}}$$

where *ℽ*_*i*_ is the MaxN for the *i*th observation, and *βx*_*i*_ is the estimated effect of the TKratio. Area was also included as a random variable. Model expansion and selection thereafter were done using Akaike information criterion (AIC).

The same statistical procedure was applied to fish sizes, represented as mean length for the species at each station. Models using different combinations of possible explanatory variables, such as depth, family, and area, in addition to the turf‐kelp ratio were done, and final model selection was done using the AIC. Analysis of the data and generation of graphs were done using R (R Core Team 2024) (ggplot2, mgcv,dplyr). Abundance and TS from the echograms were analyzed using LSSS (Large Scale Survey System) (Korneliussen et al. 2016).

## 3 Results

### 3.1 Fish Abundances and Size in Turf and Kelp Habitats Assessed by BRUV

Ten fish families dominated by labrids, gadids, and gobies were identified from baited remote underwater stereo video (BRUV, Appendix 2: Figure S2). The most abundant species were small goldsinny wrasse ( *Ctenolabrus rupestris* ) and corkwing wrasse ( *Symphodus melops* ) but there were also observations of larger Ballan wrasse ( *Labrus bergylta* ) and cuckoo wrasse ( *Labrus mixtus* ), pollock ( *Pollachius virens* ), with variable observations of poor cod ( *Trisopterus minutus* ), whitting ( *Merlangius merlangus* ), pollock ( *Pollachius pollachius* ), haddock ( *Melanogrammus aeglefinus* ), cod ( *Gadus morhua* ), *Pomatoschistus flavescens, Gobiusculus flavescens, Pomatoschistus minutus, Gobius niger, Pomatoschistus microps*, and *Aphia minuta* .

**FIGURE 2:**
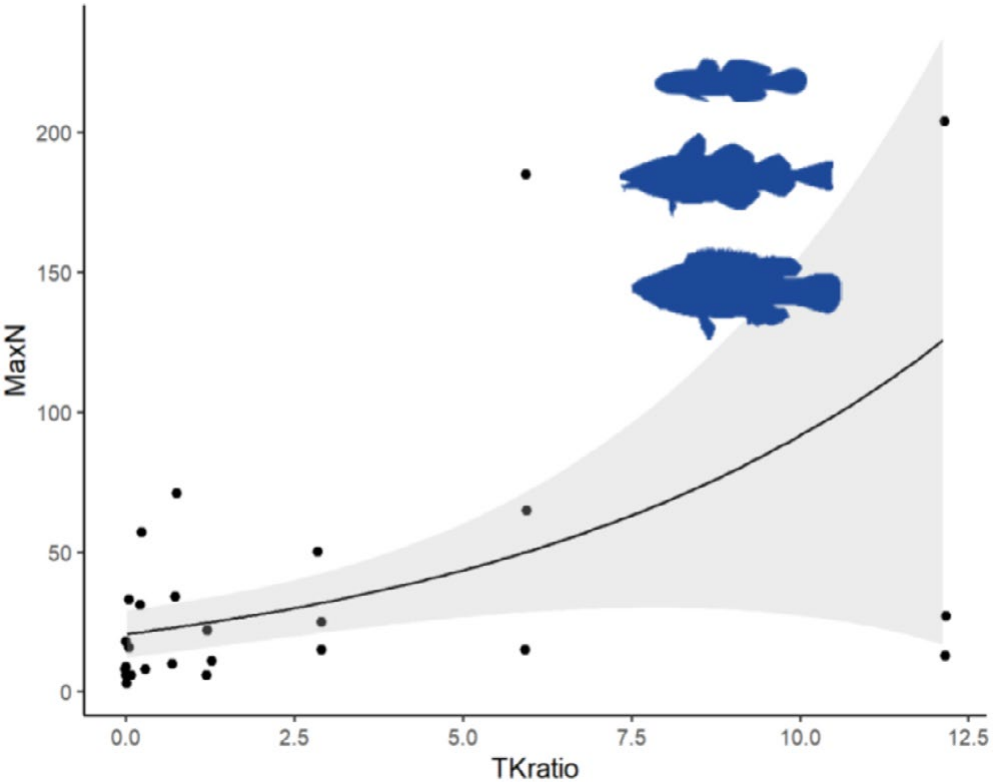
Abundances (MaxN) gobies, gadids, and labrids measured from stereo video BRUVs, (*y* axis) versus the ratio of turf to kelp (*x*‐axis) (Each point in the graph is a one‐hour BRUV observation unit).

Turf (tested as the ratio of turf: kelp) had a significant positive effect on the abundance of fish, i.e., MaxN increased with turf cover (Figure 2, Table 1, Appendix S1: Table S2). The best model also included fish families, which is best explained as tendencies of aggregation. The sites with the highest abundances had the highest turf cover and most gadids observed.

**TABLE 1:** Generalized linear model (GLM) for differences in fish abundances (response) according to turf/kep ratio (TKratio) from different families, from stereo video (BRUV, significance level marked * for the model with the lowest AIC) (significant *p*‐values in bold text). Glmer (MaxN ~ TKratio, family = negbin).

	Estimate	SE	*z*	*p*
Intercept	3.02462	0.20866	14.496	**<** **2** **e**‐**16** ***
TKratio	0.14918	0.04342	3.434	**0.000591** ***

* *p* < 0.05.

** *p* < 0.01.

*** *p* < 0.001.

**FIGURE 3:**
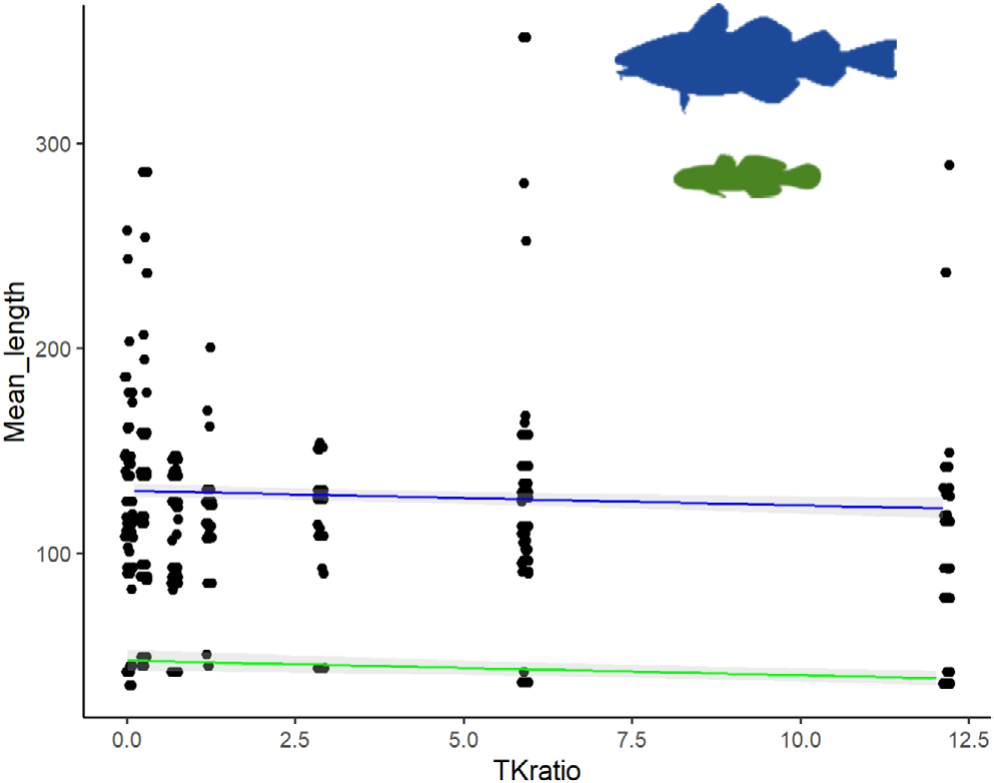
Mean length (in millimeters) for the families that showed a significant *r* (gadidae = blue, Gobiidae = green) measured from stereo video BRUVs versus the ratio of turf to kelp (*x*‐axis).

Fish size (measured as length from stereo BRUVs) was significantly smaller with increasing cover of turf relative to kelp (*p* = 0.00428), and with fish family also being a significant factor demonstrating size truncation in addition to a general size effect (Figure 3, Table 2, Appendix S1: Table S3). Gadids, labrids, and gobies were included in the model since they are the most abundant families observed, while other families being observed sporadically introduce noise and were removed. Of these three families, gadids and gobies showed a significant response. The mean length observed for gadids was 47.5 cm while gobies was 13.0 cm.

**TABLE 2:** Generalized linear model (GLM) with the lowest AIC for differences in fish length within families of fish from stereo video (BRUVs, significant *p*‐values in bold text). Generalized linear model (GLM) for differences in fish length within families of fish from stereo video (significant *p*‐values with *). Mean length ~ TKratio + Family + Area.

	Estimate	SE	*t*	*p*
(Intercept)	130.4548	1.7027	376.617	< 2e‐16***
TKratio	−0.7123	2.2487	−2.864	0.00428**
FamilyLabridae	−2.3126	2.6912	−0.859	0.39038
FamilyGobiidae	−82.9381	2.4861	−33.361	< 2e‐16***

* *p* < 0.05.

** *p* < 0.01.

*** *p* < 0.001.

### 3.2 Fish Abundances and Size in the Water Column Assessed by WBAT

**FIGURE 4:**
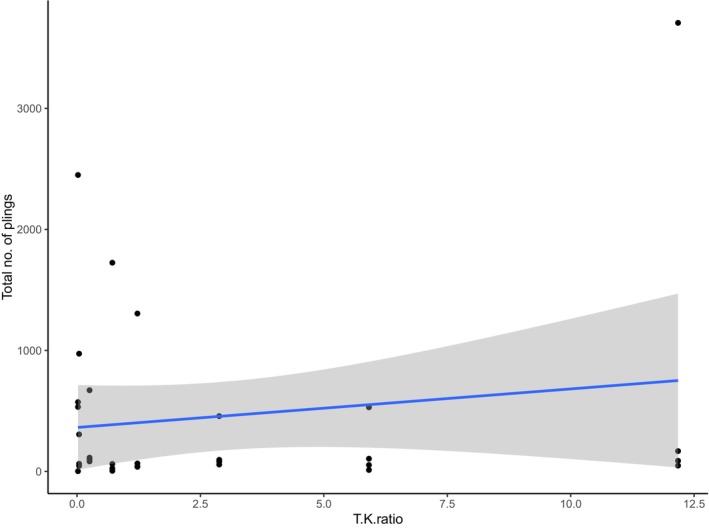
Abundances of fish from echograms (defined as echoes −20 to −60 dB) according to the ratio turf to kelp (*x*‐axis) recorded by upward facing echosounders at 10 m depth.

There were no significant differences in the abundances of fish in the water column from before sunset to after sunrise (from 20:00 to 0 08:00) at sites dominated by turf compared to sites dominated by kelp, according to GLM for echograms from bottom‐mounted echosounders (WBAT, Figure 4, Table 3, Appendix S2: Figure S3).

**TABLE 3:** GLM of abundance of fish according to turf and kelp cover and from echograms recorded by upward facing echosounders at 10 m depth (significant *p*‐values in bold text).

	Estimate	SE	*T*	*p*
Intercept	6.302e‐03	2.431e‐03	2.592	**0.0152** *
Turf:Kelp ratio	−3.986e‐05	4.772e‐05	−0.835	0.4109

* *p* < 0.05.

** *p* < 0.01.

*** *p* < 0.001.

A significant positive response for fish size (measured as TS from echogrammes) was found in the water column from before sunset to after sunrise (from 20:00 to 0 08:00) at sites dominated by turf compared to sites dominated by kelp according to GLM (Table 4, Appendix S2: Figure S3), suggesting the average size of fish was larger in the water column during the night at sites dominated by turf.

**TABLE 4:** GLM of target strength (TS) as a measure of fish size according to turf and kelp cover and from echograms recorded by upward facing echosounders at 10 m depth (significant *p*‐values in bold text).

	Estimate	SE	*T*	*p*
*TSC*				
Intercept	2.005e‐02	2.717e‐05	738.04	**< 2e‐16** ***
Turf:kelp cover	5.458e‐05	4.345e‐06	12.56	**< 2e‐16** ***

* *p* < 0.05.

** *p* < 0.01.

*** *p* < 0.001.

## 4 Discussion

**FIGURE 5:**
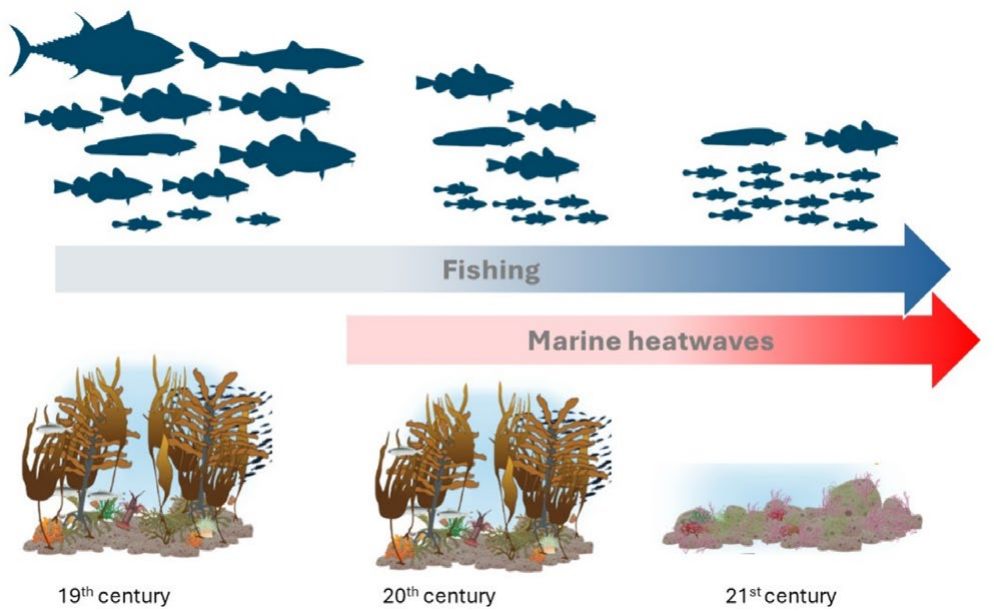
Transformation of the coastal ecosystem. Historically (the 19th century and before) the coastal ecosystem was dominated by abundant large predatory fish and kelp forests, but by the end of the 20th century, large bodied predatory fish were absent due to overfishing, causing a release of mesopredators (Svedäng and Bardon 2003; Cardinale and Svedäng 2004; Sundelöf et al. 2013, Cardinale et al. 2017). Increasing intensity and frequencies of Marine heatwaves is replacing kelp forests with turf in the 21st century (Filbee‐Dexter et al. 2020), a type of habitat favoring small‐bodied fish (this study). Kelp and turf illustration from Filbee‐Dexter and Wernberg (2018). (Image credits: Dogfish: Ignacio Contreras https://creativecommons.org/licenses/by/3.0/; Cod, wolf fish, gobies, tuna https://creativecommons.org/publicdomain/zero/1.0/. All images are downloaded from phylopic.org).

Understanding how historical and future drivers in combination impact ecosystems is vital to predict ecosystem recovery potential (Nash et al. 2016). By using non‐destructive visual and acoustic methods and comparing habitats, we showed how the transformation of coastal habitats, when important foundation species kelps are lost, may propagate through the food web, impacting associated fish abundances and sizes. The replacement of kelp by turf algae, driven by accelerating MHW, exacerbated effects in terms of size truncation in coastal fish communities, with many stocks suffering from at least a century of overfishing (Figure 5). Synnes et al. (2023) compared fish communities in our study area (Bolærne) to a zoned coastal seascape containing marine protected areas (MPA), including a no‐take zone, and found a general lack of large‐bodied piscivorous predators in areas open to fishing. In their study, this was linked to a lack of targeted management, overfishing, and subsequent predator release of smaller mesopredator species. The difference in body size of Atlantic cod (significantly larger in the Tvedestrand fjord seascape further west) was attributed to lowered fishing mortality in the zoned seascape, as compared to the outer Oslo fjord, where very few cod above the minimum legal size were found (40 cm TL). Separated by approximately 150 km and similarly exposed to the coastal current and the Skagerrak, the authors found little support for alternative hypotheses, such as e.g., putative differences in recruitment or settlement success between the areas, or stochastic processes.

The mutual but opposite‐directed effects from top‐down forcing by fishing in combination with bottom‐up forcing by MHWs may have amplifying and potentially devastating effects in coastal food webs, causing shifts that are hard to reverse: the formation of thick carpets of turf covering the sea floor during MHWs makes it difficult for kelp spores to settle and grow and thereby serves as negative feedback for kelp recovery after MHW events (Filbee‐Dexter and Wernberg 2018). Consequently, MHWs may contribute to locking the habitat into a degraded state dominated by small species and thereby undermine management efforts to restore stocks of large predatory fish that depend on or benefit from kelp forests during ontogeny.

Our results suggest, however, that these shifts act differently on fish living there depending on their relation to kelp as a habitat. Gobies, including *Pomatoschistus flavescens, Gobiusculus flavescens, Pomatoschistus minutus, Gobius niger*, and *Pomatoschistus microps* , live cryptically among algae near the sea floor (Fishbase.se, Potts et al. 1990; Goatley and Brandl 2017). By feeding on benthic invertebrates and being prey for larger fish, they have an important ecological function by transferring energy efficiently higher up in the food chain (Goatley and Brandl 2017). Reduced habitat complexity may increase encounter rates for mates and reproduction success, selecting for larger individuals (Myhre et al. 2013). Our finding of smaller gobies in turf compared to kelp may be an indication of higher predation rates and turnover in turf.

Juvenile gadids hide among kelp while adults in groups ( *Trisopterus minutus* and *Merlangius merlangus* ) or individual roving adults (*Pollachius pollachius, Melanogrammus aeglefinus*, *Pollachius virens, Gadus morhua*) use kelp forests as feeding grounds (Norderhaug et al. 2005). Cod are among the predators moving into kelp forests from the deep during the night to feed (Freitas et al. 2021), and especially large females swim large distances (Olsen et al. 2023). We did not find lower abundances of fish in the water column from before sunset to after sunrise, and the average size of fish was even larger at sites dominated by turf than kelp. Turf thus seemed to be attractive hunting grounds for visual predators, and small fish avoided the water column in open turf.

Loss of predator functions at high food web levels by fishing has been shown to impact low food web levels in coastal ecosystems by trophic cascades (Myers and Worm 2003). When top predator functions are lost, small fish such as gobies are expected to increase in abundance at the expense of grazing invertebrates they prey on and thereby allow turf to escape from grazing (Moksnes et al. 2008; Baden et al. 2012; Goatley and Brandl 2017; Pessarrodona et al. 2021). Trophic downgrading and cascading perturbations have been demonstrated in many coastal ecosystems, including macrophyte communities in the Skagerrak (e.g., Moksnes et al. 2008; Baden et al. 2012) and throughout the North Atlantic (Östman et al. 2016). Our findings that turf seemed to be equally attractive hunting grounds for roving predatory fish may have indicated how habitat properties are interfering with such trophic interactions.

An important implication of our findings is that rebuilding depleted coastal ecosystems requires management and restoration at all food web levels. Strong evidence lends support to significant improvements of target species by using large, well‐regulated and enforced MPAs for stock rebuilding purposes when environmental conditions are favorable (Canty et al. 2024). Long‐term protection of predators in New Zealand marine reserves has conferred kelp forest stability (Peleg et al. 2023). Our study demonstrates further the importance of accounting for the availability of habitats for different life stages when managing benthic fish stocks. MPAs are only partly an effective restoration tool for kelp habitats, but when the conditions are favorable, successful restoration of kelp can also be expected from nature‐based and active restoration (Parsons et al. 2004; Fredriksen et al. 2020; Earp et al. 2024; Filbee‐Dexter et al. 2024). The results from our and Synnes et al.'s (2023) study therefore suggests that a combination of active and passive restoration efforts directed to target species, accounting for MHWs and other environmental conditions, the habitats they depend on, and their states might increase the success rate of rebuilding coastal ecosystems.

The results reported herein support the notion that a ubiquitous emerging human driver (climate change) exacerbates effects from the main historical driver (overfishing) with potentially negative consequences for coastal fish communities, including species with high ecological, cultural, and economic importance. Moreover, our results highlight the importance of maintaining and restoring habitats and ecosystem functions for successfully rebuilding fish stocks, strongly pointing to the need for a holistic and ecosystem‐based management approach that accounts for the life histories of species. While climate change is hard to reverse, actions to rebuild habitats with important ecological functions for different life stages of coastal fish should have high priority in the management of coastal ecosystems.

## Author Contributions

**Kjell Magnus Norderhaug:** conceptualization (lead), funding acquisition (lead), investigation (lead), methodology (lead), project administration (lead), resources (lead), writing – original draft (lead), writing – review and editing (lead). **Portia Joy Nillos Kleiven:** data curation (equal), formal analysis (lead), visualization (lead), writing – original draft (equal), writing – review and editing (equal). **Thomas Wernberg:** investigation (equal), writing – original draft (equal), writing – review and editing (equal). **Ann‐Elin Synnes:** validation (equal), writing – review and editing (equal). **Karen Filbee‐Dexter:** investigation (equal), visualization (equal), writing – review and editing (equal). **Sigurd Espeland:** data curation (equal), formal analysis (equal), visualization (equal), writing – review and editing (equal). **Jonas Thormar:** investigation (equal), visualization (equal), writing – review and editing (equal). **Kyrre Heldal Kartveit:** data curation (equal), formal analysis (equal), methodology (equal), writing – review and editing (equal). **Lene Christensen:** data curation (equal), formal analysis (equal), software (equal), writing – review and editing (equal). **Even Moland:** conceptualization (equal), funding acquisition (equal), methodology (equal), writing – original draft (equal), writing – review and editing (equal).

## Conflicts of Interest

The authors declare no conflicts of interest.

## Supporting information


Appendix S1

## Data Availability

Data are provided in Appendix S1 and S2 and will be permanently archived and made available in Dryad if the paper is accepted for publication.
