# Supplementary material for: More but Smaller: Marine Heatwaves Exacerbate Size Truncation in Overfished Fish Communities in the Skagerrak
Source: Ecol Evol. 2025 May 8;15(5):e71404. doi: 10.1002/ece3.71404 (PMC12061468; doi:10.1002/ece3.71404)
Supplement: Supplementary file 1 — Appendix S1 [file ECE3-15-e71404-s001.docx]

# Appendix S1

Table S1. Sampling scheme showing sites, latitiude and longitude, equipment baited remote underwater stereo-video (BRUV) and Autonomous scientific echosounders (Simrad WBAT EK80), deployment date, start time for, stop time and deployment depth.

| **Site** | **Latitude** |  | **Longitude** | **Equipment** | **Date** | **T_start_** | **T_stop_** | **Depth** |
| --- | --- | --- | --- | --- | --- | --- | --- | --- |
| Langholmen | 59.1920 |  | 10.5664 | BRUV | 13.08.2020 | 15.41 | 16.41 | 5.2 |
|  |  |  |  | BRUV | 10.08.2020 | 11.45 | 12.45 | 12 |
|  |  |  |  | BRUV | 13.08.2020 | 21.51 | 22.51 | 5.5 |
|  |  |  |  | BRUV | 14.08.2020 | 21.13 | 22.13 | 16 |
|  |  |  |  | WBAT 1 | 10.08.2020 | 20.00 | 08.00 | 10 |
| Klauver | 59.1859 |  | 10.5605 | BRUV | 14.08.2020 | 18.25 | 19.25 | 5.0 |
|  |  |  |  | BRUV | 10.08.2020 | 12.32 | 13.32 | 9.0 |
|  |  |  |  | BRUV | 11.08.2020 | 21.43 | 22.43 | 5.5 |
|  |  |  |  | BRUV | 11.08.2020 | 21.46 | 22.46 | 15 |
|  |  |  |  | WBAT 2 | 10.08.2020 | 20.00 | 08.00 | 10 |
| Tova | 59.1818 |  | 10.5929 | BRUV | 10.08.2020 | 16.52 | 17.52 | 7 |
|  |  |  |  | BRUV | 10.08.2020 | 22.53 | 23.53 | 6.0 |
|  |  |  |  | BRUV | 10.08.2020 | 17.01 | 18.01 | 12 |
|  |  |  |  | BRUV | 10.08.2020 | 22.56 | 23.56 | 13 |
|  |  |  |  | WBAT 1 | 12.08.2020 | 20.00 | 08.00 | 10 |
| Østre Bolærne | 59.2093 |  | 10.5679 | BRUV | 11.08.2020 | 18.05 | 19.05 | 5.5 |
|  |  |  |  | BRUV | 11.08.2020 | 18.05 | 19.05 | 15 |
|  |  |  |  | BRUV | 10.08.2020 | 23.11 | 00.11 | 5.0 |
|  |  |  |  | BRUV | 12.08.2020 | 21.40 | 22.40 | 13 |
|  |  |  |  | WBAT 2 | 11.08.2020 | 20.00 | 08.00 | 11 |
| Fulehuk | 59.1720 |  | 10.6000 | BRUV | 13.08.2020 | 13.31 | 14.31 | 5.0 |
|  |  |  |  | BRUV | 13.08.2020 | 13.39 | 14.39 | 18 |
|  |  |  |  | BRUV | 13.08.2020 | 21.05 | 22.05 | 5.5 |
|  |  |  |  | BRUV | 13.08.2020 | 21.20 | 22.10 | 16 |
|  |  |  |  | WBAT 2 | 13.08.2020 | 20.00 | 08.00 | 10 |
| Skarvesetet | 59.1911 |  | 10.5808 | BRUV | 12.08.2020 | 14.51 | 15.51 | 5.0 |
|  |  |  |  | BRUV | NA | NA | NA | NA |
|  |  |  |  | BRUV | 14.08.2020 | 21.20 | 22.20 | 6.0 |
|  |  |  |  | BRUV | 14.08.2020 | 21.13 | 22.13 | 16 |
|  |  |  |  | WBAT 2 | 12.08.2020 | 20.00 | 08.00 | 10 |
| Rauer | 59.1752 |  | 10.5783 | BRUV | 12.08.2020 | 14.41 | 15.41 | 5.8 |
|  |  |  |  | BRUV | 12.08.2020 | 14.43 | 15.43 | 15 |
|  |  |  |  | BRUV | 13.08.2020 | 21.00 | 22.00 | 5.4 |
|  |  |  |  | BRUV | NA | NA | NA | NA |
|  |  |  |  | WBAT 1 | 13.08.2020 | 20.00 | 08.00 | 10 |
| Garnholmen | 59.2038 |  | 10.5775 | BRUV | 11.08.2020 | 18.15 | 19.15 | 5.2 |
|  |  |  |  | BRUV | 14.08.2020 | 13.50 | 14.50 | 12 |
|  |  |  |  | BRUV | 12.08.2020 | 21.10 | 22.10 | 5.8 |
|  |  |  |  | BRUV | 12.08.2020 | 21.17 | 22.17 | 13 |
|  |  |  |  | WBAT 1 | 11.08.2020 | 20.00 | 08.00 | 10 |

# Table S2. Model selection for GLM models with fish abundance (MaxN) as a response variable of a combination of different predictor variables. The first two annotations with (1|variable) indicates that the variable was factored in as random effect.

| Model | df | AIC |
| --- | --- | --- |
| MaxN ~ TKratio + (1\|Area) +(1\|Family) | 4 | 2307.961 |
| MaxN ~ TKratio + (1\|Area) | 3 | 1462.1530 |
| **MaxN ~TKratio + Family** | **11** | **720.1789** |
| MaxN ~ TKratio + Area | 8 | 1445.7676 |
| MaxN ~ TKratio | 2 | 1502.4180 |
| MaxN ~ Area | 8 | 1445.7676 |
| MaxN ~ Family | 10 | 1037.9184 |

# Table S3. Model selection for GLM models with fish size) as a response variable of a combination of different predictor variables. The first two annotations with (1|variable) indicates that the variable was factored in as random effect.

| Model | Intercept p value | TKratio p value | AIC |
| --- | --- | --- | --- |
| **Mean length ~ TKratio + Family + Area** | 0.09305 (.) | 0.63988 | **8463** |
| Mean length ~ TKratio | 2 x 10^-16^  (***) | 2 x 10^-16^  (***) | 9875.3 |
| Mean length ~ TKratio + Area | 0.231 | 0.800 | 9759.8 |
| Mean length ~ TKratio + Family | 2 x 10^-16^  (***) | 7.43 x 10^-16^ | 8519.9 |
| Mean length ~ TKratio + Family + (1\|Area) | 2 x 10^-16^  (***) | 0.126510 | 8469.73 |
| Mean length ~ TKratio + (1\|Family + (1\|Area) | 2 x 10^-16^  (***) | 0.113 | 8486.4 |
| Mean length ~ TKratio + (1\|Family) | 3.357 (t-value) | -2.808 (t- value) | 9261.59 |
| Mean length ~ TKratio + (1\|Area) | 16.382 (t- value) | -3.325 (t- value) | 10041.49 |

# Appendix S2


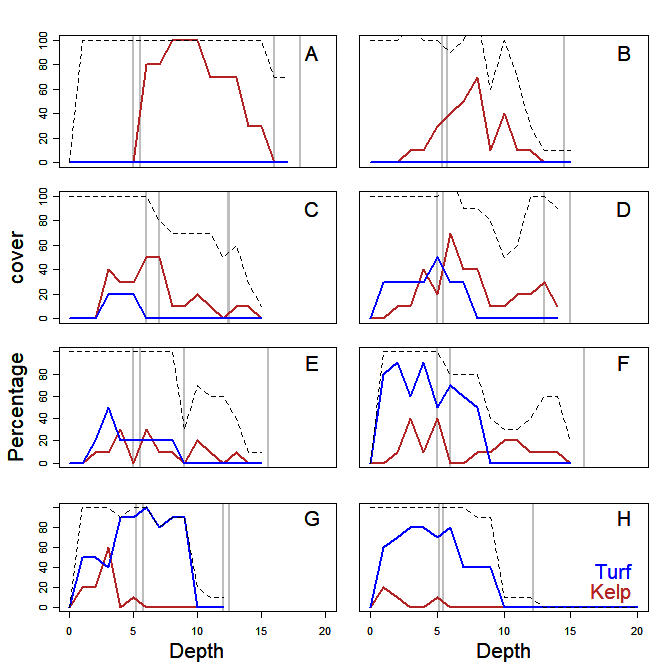


Figure S1. The vertical distribution of kelp and turf. The blue line is percentage cover of turf, red is kelp (K) at different depths given on the x-axis. The dotted line is the combined total cover of vegetation. The vertical lines represent depths where videos have registered fish at each location. Locations are sorted from highest kelp cover to lowest kelp cover: A) Fulehuk, B) Rauer, C) Tova, D) Bolærne, E) Klauer, F) Skarvesetet, G) Garnholmen, H) Langholmen. Grey vertical lines shows BRUV observation depths.


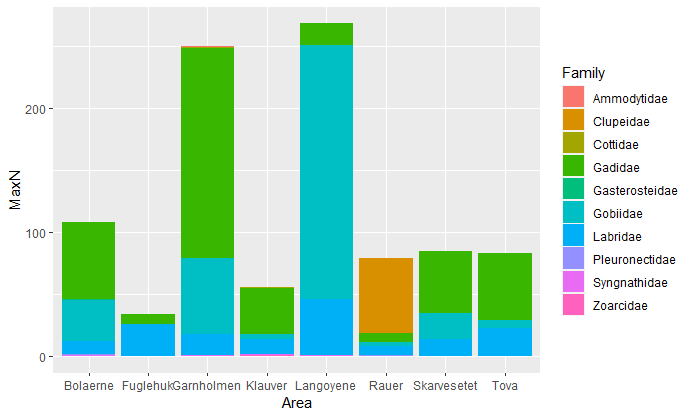


Figure S2. Relative numbers per fish family recorded per area in 1 hour using baited remote underwater stereo-video (BRUV).


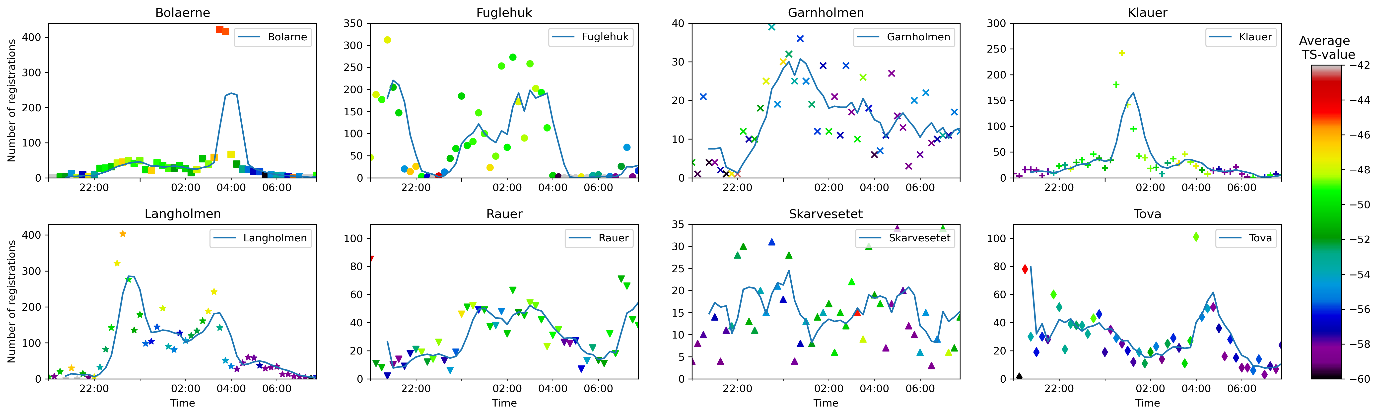


Figure S3. Abundances of fish (line and left axis, n/m^3^) and target strength (TS colour, right axis) through the day from echograms at all sites.
